# Red Quinoa Bran Extract Prevented Alcoholic Fatty Liver Disease via Increasing Antioxidative System and Repressing Fatty Acid Synthesis Factors in Mice Fed Alcohol Liquid Diet

**DOI:** 10.3390/molecules26226973

**Published:** 2021-11-18

**Authors:** Ting-An Lin, Bo-Jun Ke, Shih-Cheng Cheng, Chun-Lin Lee

**Affiliations:** 1 Department of Life Science, National Taitung University, Taitung 950, Taiwan; tingan81@gmail.com (T.-A.L.); kebojun@hotmail.com (B.-J.K.); 2 Sinfong Agricultural Technology Company, Taipei 110, Taiwan; wacau88@yahoo.com.tw

**Keywords:** *Chenopodium formosanum*, red quinoa bran, alcoholic liver disease, red quinoa extraction process, rutin

## Abstract

Alcohol is metabolized in liver. Chronic alcohol abuse results in alcohol-induced fatty liver and liver injury. Red quinoa (*Chenopodium formosanum*) was a traditional staple food for Taiwanese aborigines. Red quinoa bran (RQB) included strong anti-oxidative and anti-inflammatory polyphenolic compounds, but it was usually regarded as the agricultural waste. Therefore, this study is to investigate the effect of water and ethanol extraction products of RQB on the prevention of liquid alcoholic diet-induced acute liver injury in mice. The mice were given whole grain powder of red quinoa (RQ-P), RQB ethanol extract (RQB-E), RQB water extract (RQB-W), and rutin orally for 6 weeks, respectively. The results indicated that RQB-E, RQB-W, and rutin decreased alcoholic diet-induced activities of aspartate aminotransferase and alanine aminotransferase, and the levels of serum triglyceride, total cholesterol, and hepatic triglyceride. Hematoxylin and eosin staining of liver tissues showed that RQB-E and RQB-W reduced lipid droplet accumulation and liver injury. However, ethanol extraction process can gain high rutin and antioxidative agents contents from red quinoa, that showed strong effects in preventing alcoholic fatty liver disease and liver injury via increasing superoxide dismutase/catalase antioxidative system and repressing the expressions of fatty acid synthesis enzyme acetyl-CoA carboxylase.

## 1. Introduction

The liver is an important organ for metabolism and detoxification. Chronic liver injury is usually followed by steatosis, hepatitis, cirrhosis, and liver cancer [1]. Long-term alcohol consumption weakens the activities of anti-oxidative enzymes, causes lipid peroxidation and alcoholic liver disease (ALD) [2]. Fatty liver is a primary syndrome during ALD. Previous research has shown that patients with ALD are a high-risk group to get hepatic fibrosis and cirrhosis [3]. In the liver, alcohol metabolism involves three enzymes: alcohol dehydrogenase (ADH), cytochrome P-450 2E1 (CYP2E1), and catalase (CAT) [4]. Alcohol is converted to acetaldehyde by ADH, and then it induces CYP2E1 to release reactive oxygen species (ROS). This process damages the mitochondrion and results in oxidative stress in the liver [5]. The free radicals damage DNA, proteins, and lipids in the cells. Oxidative stress is the primary factor to cause ALD [6]. Moreover, long-term alcohol consumption results in inflammation and oxidative stress in the liver. It also inhibits the activities of 5′ adenosine monophosphate-activated protein kinase (AMPK), peroxisome proliferator-activated receptor alpha (PPAR-α), carnitine palmitoyltransferase I (CPT-1), and acetyl-CoA carboxylase (ACC). By contrast, it increases the level of lipid peroxidation. A high level of activity of the sterol regulatory element-binding protein (SREBP) reduces lipolysis and increases fatty acid synthesis [6,7]. Therefore, large amount of lipid accumulates in the liver and results in fatty liver. Fatty liver is reversible and it is an indication of the early stage of ALD.

In this decade the focus on natural products and their bioactive compounds have been on nutrition and health care, especially food supplements for liver health, regulation of blood lipids, and immune regulation [8]. *Chenopodium formosanum* known as red quinoa is a native plant in Taiwan [9]. Taiwanese aborigines have harvested red quinoa for hundreds of years. Red quinoa includes high contents of betanin, protein, vitamins, essential amino acids, and minerals [9]. Furthermore, red quinoa contains about 14 g of dietary fiber per 100 g. Red quinoa dietary fiber helps to reduce constipation, reduces the level of cholesterol [10], and prevents colon cancer [11]. Previous research reported that red quinoa (RQ) contains multiple polyphenolic compounds including rutin, betanin, and kaempferol [12]. Rutin, the main bioactive compound in the bran of RQ (RQB), has the effects of anti-oxidation [13], anti-inflammation, [14] anti-allergy [15], and hepatic protection [16]. In the previous study, the polyphenol including quercetin, quercetin-3-glucoside, and rutin were proven to demonstrate protective effect on alcohol-induced damage in hepatocytes by anti-oxidation and anti-inflammation [17]. RQB is a good source for rutin and other polyphenols or natural phenols. It has the potential to eliminate free radicals and has a protective effect on the liver through anti-oxidation and anti-inflammation. In this study, C57BL/6J mice was induced ALD and liver injury by a liquid ethanol diet and used to research the effect of red quinoa whole grain powder, RQB ethanol extract (RQB-E), RQB water extract (RQB-W), and rutin on the prevention of fatty liver and liver damage.

## 2. Results

### 2.1. The Quantity of Rutin in Red Quinoa and the Extract Ratio

The RQB was extracted with water for the preparation of RQB-W, or extracted with 50% ethanol for the preparation of RQB-E. Regarding the extract ratio (Table 1), water extraction and 50% (*v/v*) ethanol extraction can yield 19.70% and 14.87% extract ratio (*w/w*) of the RQB, respectively. However, RQB-E contained 10.65 ± 1.34 mg of rutin and RQB-W contained 2.45 ± 0.82 mg of rutin. The concentration of rutin in RQB-E was about 4.3 times that of rutin in RQB-W.

### 2.2. Body Weight, Liver Weight and Liver Weight/Body Weight Ratio

Alcohol consumption can cause abnormal lipid metabolism, and then result in TG accumulation in the liver [18,19]. We measured the relative liver to body weight to evaluate the level of liver injury. In Table 2, the EtOH group had significantly lower body weight (*p* < 0.05) than the NOR group. Although the EtOH group had significantly lower liver weight (*p* < 0.05) than the NOR group, this result still indicated that the EtOH group had significantly higher ratio of liver weight to body weight (*p* < 0.05). The RQB-E, RQB-W, and Rutin group had significantly lower ratio of liver weight to body weight than the EtOH group (*p* < 0.05) but the RQ-P group showed no significant difference (*p* > 0.05).

### 2.3. Serum AST, ALT, and ALP Activities

The activities of AST and ALT in serum are important indices indicating liver function [20]. The activities of AST, ALT, and ALP in the EtOH group were significantly higher than in the NOR group (*p* < 0.05). This result showed that the liquid ethanol treatment successfully induced liver injury in mice. The RQ-P group, RQB-E, RQB-W, and Rutin groups all had significantly lower AST, ALT, and ALP activities, compared to the EtOH group (*p* < 0.05). The RQB-E group has the most effect of reducing the ALT activities increased by liquid ethanol treatment among all groups (Table 3).

### 2.4. Serum and Hepatic Lipid Contents

Ethanol and acetaldehyde break the balance of redox reactions. They generate a lot of NADH and interrupt the metabolism in cells. This process inhibits fatty acid oxidation and promotes fatty acid synthesis, and then results in fatty liver [21]. The levels of serum TC and TG in the EtOH group were significantly higher than in the NOR group (*p* < 0.05). This result shows that ethanol diet increased the levels of blood lipids. However, all of the samples showed significantly lower levels of TC and TG in serum, as compared to the EtOH group (*p* < 0.05). In the liver, the level of TG and glycerol in the EtOH group were higher than in the NOR group, but the level of TC in the EtOH group showed no difference when compared to the NOR group (*p* > 0.05). RQB-E and RQ-P showed significantly decreased levels of TG and glycerol in the liver, compared to the EtOH group (*p* < 0.05) (Table 4).

### 2.5. Hepatic Pathological Changes

**Figure 1:**
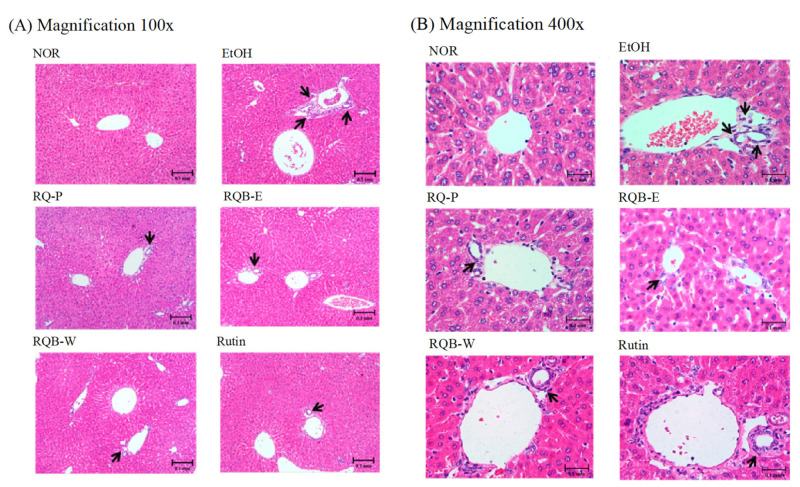
Hepatic pathological changes of the AFLD mice. (**A**) Magnification 100×, (**B**) magnification 400× (black arrows indicate the position of liver injury). Two groups of mice were fed with normal liquid diet (NOR group) or ethanol liquid diet (EtOH group) without the administration of test materials, respectively. The other mice fed ethanol liquid diet were administrated with red quinoa powder (5.13 g/kg B.W./day, RQ-P group), red quinoa bran ethanol extracts (1.54 g/kg B.W./day, RQB-E group), red quinoa bran water extracts (1.54 g/kg B.W./day, RQB-W group), and rutin (16.4 mg/kg B.W./day, Rutin group).

The animals were euthanized and the liver tissue was fixed and stained by hematoxyline and eosin. Figure 1 indicated the hepatic pathological changes in the 100× and 400× magnification. The liver section in the EtOH group was observed to have microvesicula steatosis and cell swelling (as indicated by the black arrow). The liver sections in the RQ-P group, RQB-W and Rutin group were observed to have slight macrophage infiltration near the central vein and little lipid accumulation. However, the RQB-E group showed no difference to the NOR group. Higher rutin and other polyphenol contents probably contributed more protection to the ethanol extract against AFLD.

### 2.6. Lipid Peroxidation in the Liver

**Figure 2:**
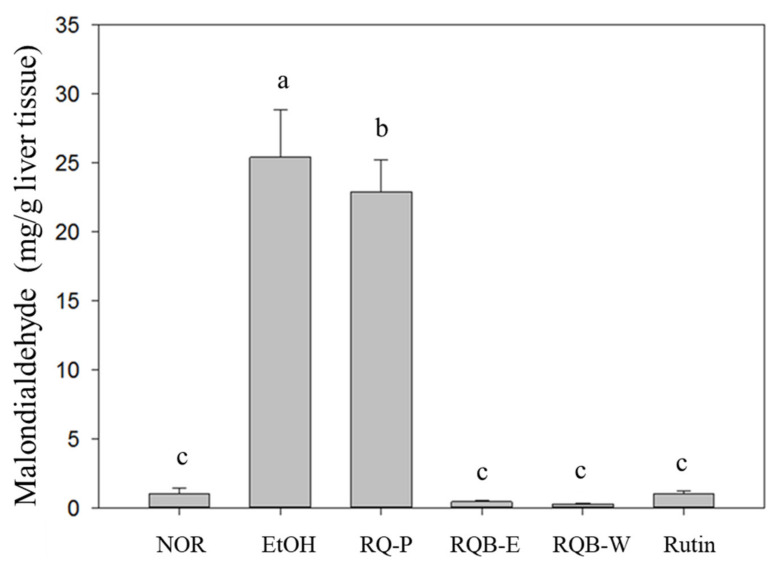
The levels of lipid peroxidation in the liver. The data are presented as the means ± SD (*n* = 8). The means followed by the same letter within each column did not differ significantly from each other (*p* > 0.05). However, the means followed by different letter expressed significant difference from each other (*p* < 0.05). Two groups of mice were fed with normal liquid diet (NOR group) or ethanol liquid diet (EtOH group) without the administration of test materials, respectively. The other mice fed ethanol liquid diet were administrated with red quinoa powder (5.13 g/kg B.W./day, RQ-P group), red quinoa bran ethanol extracts (1.54 g/kg B.W./day, RQB-E group), red quinoa bran water extracts (1.54 g/kg B.W./day, RQB-W group), and rutin (16.4 mg/kg B.W./day, rutin group). TBARS: thiobarbituric acid reactive substance.

Alcohol metabolism results in oxidative stress and promotes lipid peroxidation in the liver. Thiobarbituric acid reactive substance (TBARS) method was used to evaluate the levels of the lipid peroxidation and oxidative stress according to the formation levels of TBARS [22,23]. In the result (as shown in Figure 2), after a liquid ethanol diet intake for 6 weeks, the EtOH group had a significantly higher TBARS level, compared to the NOR group (*p* < 0.05). This result shows that long-term alcohol consumption resulted in severe lipid peroxidation. After treatment, TBARS levels of the experimental groups decreased significantly (*p* < 0.05). Significant inhibition of lipid peroxidation was found in the RQB-E, RQB-W, and Rutin groups but not in RQ-P group. Therefore, the results suggested that rutin and the other polyphenol in the water or ethanol extract may perform the inhibition. However, the whole powder may have weak effect due to the lower bio-absorption of rutin and the other polyphenol, even its rutin content is equal to the extract.

### 2.7. Activities of Antioxidative System

**Figure 3:**
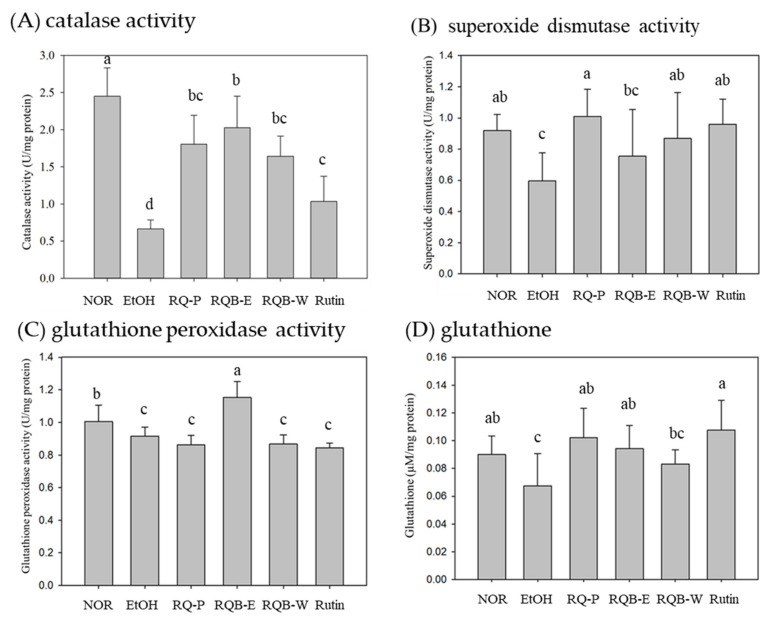
The activities of catalase (**A**), superoxide dismutase (**B**), glutathione peroxidase (**C**), and the levels of glutathione (**D**) in liver. The data are presented as the means ± SD (*n* = 8). The means followed by the same letter within each column did not differ significantly from each other (*p* > 0.05). However, the means followed by different letter expressed significant difference from each other (*p* < 0.05). Two groups of mice were fed with normal liquid diet (NOR group) or ethanol liquid diet (EtOH group) without the administration of test materials, respectively. The other mice fed ethanol liquid diet were administrated with red quinoa powder (5.13 g/kg B.W./day, RQ-P group), red quinoa bran ethanol extracts (1.54 g/kg B.W./day, RQB-E group), red quinoa bran water extracts (1.54 g/kg B.W./day, RQB-W group), and rutin (16.4 mg/kg B.W./day, Rutin group).

Oxidative stress is a primary factor inducing ALD. The high levels of ROS decrease the activities of anti-oxidative enzymes in the liver. Free radical and peroxidation damage the DNA in liver cells [7]. The activity of catalase (CAT) is shown in Figure 3A. The EtOH group had significantly lower CAT activity than the NOR group (*p* < 0.05). The samples showed significantly increased CAT activity in the RQ-P, RQB-E, RQB-W, and Rutin groups, compared with the EtOH group (*p* < 0.05). The superoxide dismutase (SOD) activity in the EtOH group was significantly lower than in the NOR group (*p* < 0.05). The RQ-P, RQB-W, and Rutin group had significantly higher SOD activity than in the EtOH group (*p* < 0.05) but the RQB-E group showed no difference compared to the EtOH group (*p* > 0.05) (Figure 3B).

Regarding the activity if glutathione peroxidase (GPx) (Figure 3C), the EtOH group showed a significantly lower level of GPx activity than in the NOR group (*p* < 0.05). Only the RQB-E group showed significantly increased GPx activity (*p* < 0.05). The level of glutathione (GSH) in the EtOH group was significantly lower than in the NOR group (*p* < 0.05). The RQ-P, ROB-E, and rutin group had significantly higher levels of GSH than in the EtOH group (*p* < 0.05) but the RQB-W group showed no difference compared to the EtOH group (*p* > 0.05) (Figure 3D).

### 2.8. Mediation for Fatty Acid Metabolism

**Figure 4:**
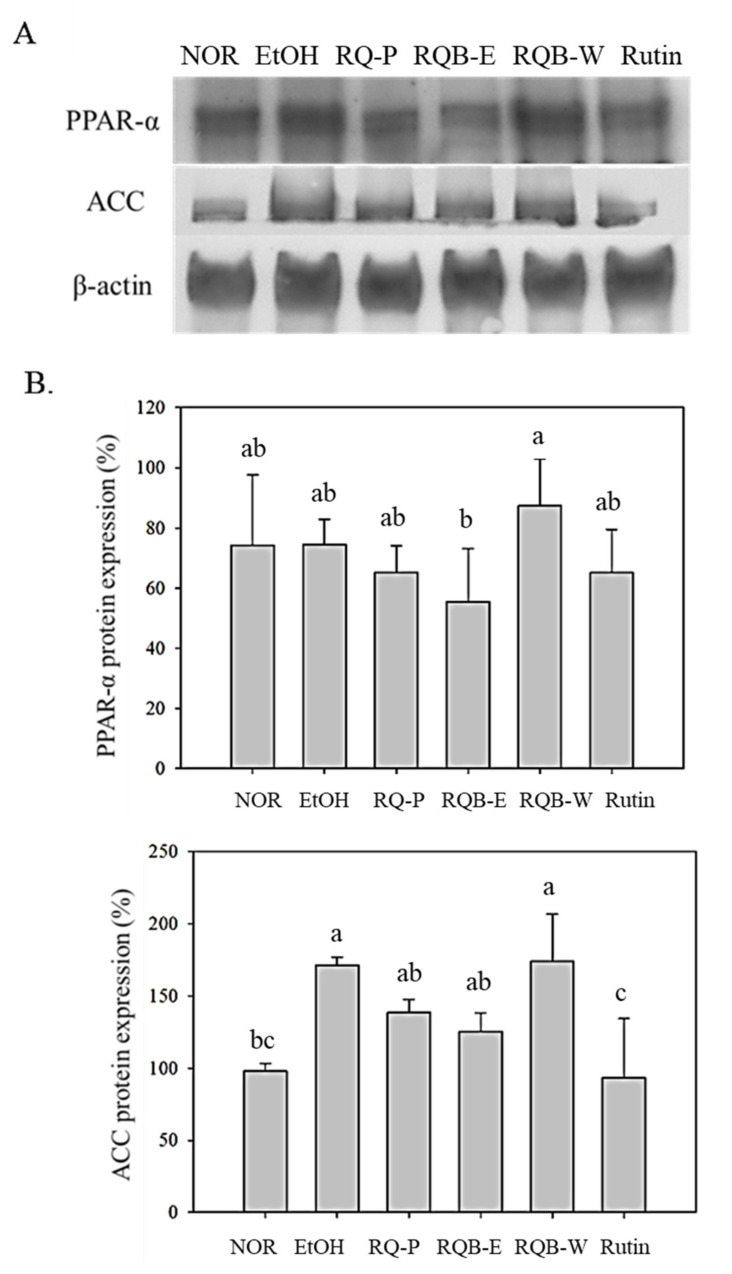
Expressions of ACC and PPAR-α in livers were detected by Western blot analysis (**A**). The levels of the proteins were subsequently quantitated by densitometric analysis (**B**). The data are presented as the means ± SD (*n* = 8). The means followed by the same letter within each column did not differ significantly from each other (*p* > 0.05). However, the means followed by different letter expressed significant difference from each other (*p* < 0.05). Two groups of mice were fed with normal liquid diet (NOR group) or ethanol liquid diet (EtOH group) without the administration of test materials, respectively. The other mice fed ethanol liquid diet were administrated with red quinoa powder (5.13 g/kg B.W./day, RQ-P group), red quinoa bran ethanol extracts (1.54 g/kg B.W./day, RQB-E group), red quinoa bran water extracts (1.54 g/kg B.W./day, RQB-W group), and rutin (16.4 mg/kg B.W./day, Rutin group). ACC: acetyl CoA carboxylase; PPAR-α: proliferator-activated receptor alpha.

AMPK and PPAR-α control the lipid metabolism and lipid synthesis in the liver. PPAR-α can mediate fatty acid metabolism by exhibiting β-oxidation of the fatty acid in the liver. PPAR-α also regulates ACC and decreases the level of TG [24,25]. In the result of Figure 4, PPAR-α expression of EtOH group had no significant difference as compared to NOR group (*p* > 0.05). All sample groups also did not significantly increase PPAR-α (*p* > 0.05), although RQB-W slightly raised PPAR-α expression without significant difference (*p* > 0.05). However, ACC expression was significantly higher in the EtOH group than in the NOR group (*p* < 0.05), indicating that the daily ethanol diet increased the ACC expression and promoted fatty acid synthesis. The RQ-P and RQ-E can slightly lower the ACC expression without significant difference (*p* > 0.05), however, its functional compound rutin can perform significant effects on lowering ACC expression (*p* < 0.05).

## 3. Discussion

*Chenopodium formosanum*, a native species in Taiwan, is called red quinoa because of its rich and diverse red, purple, and yellow pigments. Whole red quinoa includes the grain and the bran. The grains of red quinoa are regarded as health food due to the rich protein and dietary fiber. However, the bran of red quinoa used to be considered agricultural waste, however, it contains a variety of polyphenols, and rutin is one of the important functional compounds. Rutin has antioxidative [13], anti-inflammatory [14], and hepatoprotective [16], anti-allergic properties [15]. In addition, this study is based on the fact that the bran of red quinoa may be a natural antioxidant, and red quinoa grains have dietary fiber, so red quinoa has the potential to improve AFLD. This study also explored the differences in the effects of water extracts (RQB-W) and ethanol extracts (RQB-E) in improving AFLD.

In this study, the mice were fed the ethanol liquid diet for the induction of AFLD. According to the feed formula, alcohol replaces 36% of carbohydrates in the feed, and the normal diet and ethanol diet have equal calories (1000 kcal/L). Although the normal liquid diet and the liquid ethanol diet contained the same amount of energy, previous studies reported that alcohol interrupts nutrient absorption [26]. One gram of alcohol provides 7 kilocalories [27]. The liquid ethanol diet-induced liver injury mice had higher relative liver to body weight than compared to the healthy mice. Alcohol abuse caused weight loss but it leads to abnormal enlargement of the liver. However, red quinoa extracts and rutin reduced the relative liver to body weight. Dietary fiber of the red quinoa bran powder promotes digestion and absorption, increases the sense of fullness, and helps weight loss [28]. Ethanol is converted to acetaldehyde by ADH and (microsomal ethanol oxidizing system) MEOS, and then synthesizes fatty acid by acetyl-CoA pathway. TG accumulates in the liver leading to fatty liver and enlarges the liver [29,30]. It also causes higher levels of TG in the serum. The H&E staining, showed that the liquid ethanol diet-induced liver injury mice had severe lipid accumulation and macrophage infiltration. Red quinoa extracts and rutin inhibited the lipid accumulation in the liver and reduced macrophage infiltration. The activities of AST and ALT are the indicators of liver function. ALP is a key indicator of liver and gallbladder functions [31]. AST mostly is found in the heart, kidney, liver, and muscle. ALT is mostly in the liver. When the liver function is diseased or damaged, AST, ALT, and ALP will be released into the bloodstream [32]. Long-term alcohol abuse causes liver and gallbladder injuries, resulting in AST, ALT, and ALP levels being higher in the serum. Red quinoa, red quinoa extracts, and rutin can prevent AFLD.

Alcohol metabolism occurs in the liver by MEOS pathway and CAT pathway. It requires the CYP2E1 enzyme, forms ROS and leads to oxidative stress [5,7]. ROS are highly reactive chemical forms of oxygen, such as superoxide anion and hydroxyl radical. ROS damages the cells and induce lipid peroxidation [33]. However, SOD converts superoxide anions to hydrogen peroxide, and then CAT converts hydrogen peroxide to water. At the same time, GSH converts to GSSG through GPx and converts back to GSH through glutathione reductase (GR) and NADPH. Long-term alcohol abuse increased the level of lipid peroxidation. After consumption of red quinoa powder, red quinoa extracts, and rutin, the levels of lipid peroxidation decreased. Red quinoa and rutin prevented ROS-induced oxidative injury. In addition, alcohol abuse decreased SOD, CAT, and GPx activities and GSH level. However, red quinoa powder, RQB-W, and rutin increased SOD and CAT activities. RQB-E increased CAT and GPx activities and GSH level. Both RQB-E and RQB-W have antioxidant effects, but there are still differences. The reason may be due to their different composition of polyphenol compounds. In addition, RQB-E has higher rutin concentration, and RQB-W has higher water soluble dietary fiber and polyphenolic compound. However, this result proves that RQ has anti-oxidation effect.

PPAR-α stimulates fatty acid β-oxidation, and fatty acid transportation. It is a key factor to regulate fatty acid metabolism. ACC also plays an important role in fatty acid synthesis. Increasing PPAR-α expression and repressing ACC expression can modulate the fatty acid metabolism, and further block TG biosynthesis [24,25]. Alcohol metabolism increases the expression of ACC and promotes fatty acid synthesis. However, RQB-E and rutin had an inhibitory effect in the expression of ACC, but had no inhibitory effect in the expression of PPAR-α. Therefore, RQ may perform the suppression on fatty acid metabolism rather than the stimulation on fatty acid β-oxidation.

**Figure 5:**
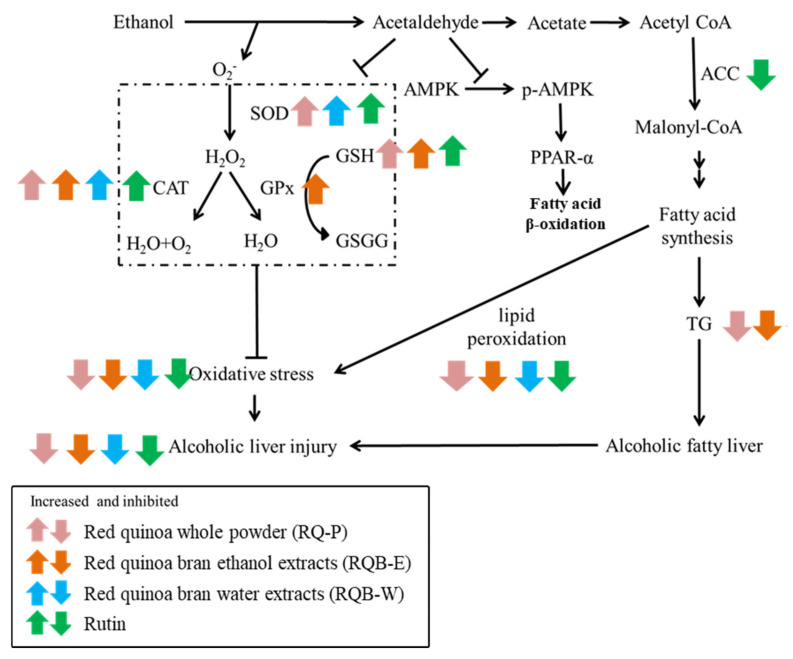
The regulation on the relative factors expression in liver of AFLD mice. The arrow indicates the increased or decreased regulation with significant effect (*p* < 0.05, as compared to EtOH group).

The possible regulation pathway of RQ-P, RQB-E, RQB-W, and rutin on the prevention of AFLD is as shown in Figure 5. RQ-P was whole grain powder including the bran and the grain had 1.65 mg/g rutin. RQ-P had more effect in lowering the liver TG content than RQB-E and RQB-W, it might be from the dietary fiber of the grain. However, the bran was proven as an important antioxidative product in the red quinoa. Regarding the extract of the bran, RQB-E (10.65 ± 1.34 mg/g rutin) contains more rutin than RQB-W (2.45 ± 0.82 mg/g), which showed that RQB-E had more liver protection potential than RQB-W. RQB-E prevented ethanol-induced oxidative stress via raising antioxidative enzyme system (CAT and GPx) and suppressing lipid peroxidation. RQB-E and rutin both inhibited the expression of ACC involved in the regulation of fatty acid biosynthesis, which should be a key reason for lowering TG accumulation in liver. Therefore, rutin can be regarded as the main bioactive polyphenolic compound in red quinoa because its regulation effect was similar to RQB-E. Therefore, this study provided an application of the bran of red quinoa on the functional food development for the prevention of AFLD.

According to the above results of this study, rutin can be confirmed as an important functional component in red quinoa. However, the previous study indicates that betanin and kaempferol were detected in the red quinoa water extract, which improved the oxidative damage induced by tert-butyl hydroperoxide (t-BHP) in HepG2 cells by increasing the GSH content, reducing ROS production and caspase-3 activity, and increasing the ratio of apoptosis index Bcl-2/Bax [34]. Quinoa seeds are also rich in vitamins in the human diet. Quinoa was also rich in vitamin E (tocopherol). The total tocopherol content of quinoa seeds ranged from 37.49 to 59.82 μg/g. All four tocopherol isoforms (α, β, γ, and δ) have been detected in quinoa seeds [35]. Tocopherols are strong antioxidants, which may also have an effect with the polyphenol compounds in red quinoa to enhance the antioxidative system.

In conclusion, liquid ethanol diet induced fatty liver, oxidative stress, and liver steatosis in the AFLD mice model. Daily feeding of RQ-P, RQB-W, and RQB-E showed an effect in lowering the levels of TC, TG, AST, ALT in serum, and the levels of pathological hepatic steatosis in AFLD mice. Furthermore, the RQB-E had more effect than RQB-W in raising antioxidation enzyme system. However, RQB-W but not RQB-E had an effect in suppressing ACC expression. Rutin should be one of the most important polyphenolic compounds in RQB-E. According to the results, red quinoa bran should no longer be treated as agricultural waste. In the whole red quinoa, the bran contains rutin and other polyphenolic compounds and the grain also contains dietary fiber, which can be recommended as a functional natural food to prevent alcoholic fatty liver and liver injury.

## 4. Materials and Methods

### 4.1. Chemicals and Reagents

The chemicals and standards including Triton X-100, Tris, NaF, SDS, deoxy-cholate, EDTA, EGTA, Na_3_VO_4,_ NaH_2_PO_4_, and rutin were purchased from Sigma-Aldrich, Co. (St. Louis, MO, USA). Ethanol (95%) was purchased from Taiwan Tobacco and Liquor Co. (Taipei, Taiwan). Folin-Ciocalteau agent and gallic acid were purchased from Panreac Quimina S.A. (Barcelona, Spain). DMSO, phenol, sulfuric acid, and sodium carbonate were purchased from Merck Co. (Darmstadt, Germany).

### 4.2. Sample Preparation

The grain and the bran of red quinoa (*Chenopodium formosanum* Koidz) were provided by Sin-Fong agricultural science and technology company (Taipei, Taiwan). The whole grain of RQ and RQB were stored at 4 °C before use. Whole RQ including grain and bran was ground into powder (RQ-P). The RQB was extracted with water for the preparation of RQB-W, or extracted with 50% ethanol for the preparation of RQB-E. Regarding the preparation of RQB-E, the RQB was extracted with 5 volumes of 50% (*v/v*) ethanol at 50 °C for 2 h. The supernatant was collected by centrifugation at 6000× *g* for 5 min at 4 °C, and then was concentrated by using rotary evaporator to remove the ethanol. After lyophilization, the dried RQB was collected for the animal test. Regarding the preparation of RQB-W, the RQB was extracted with 10 volumes of ultra-pure water at 50 °C for 2 h and then centrifuged at 6000× *g* for 5 min at 4 °C. The dried RQB-W was obtained after vacuum concentration and lyophilization. The rutin concentrations in RQ-P, RQB-E, and RQB-W were analyzed by high performance liquid chromatography (HPLC) with a reverse-phase column (Mightysil RP-18 GP 5 μm C18, 250–4.6 mm, Kanto Chemical Co., Inc., Tokyo, Japan) and photodiode array detector (DAD, L-2000 series, Hitachi, Japan). The mobile phase (0.1% trifluoroacetic acid solution: acetonitrile = 45:55) was eluted with 1.0 mL/min of flow rate and detected at 250 nm.

### 4.3. Animal Model and Grouping

Forty-eight male C57BL/6J mice (7 weeks old) were housed in plastic cages (4 mice in each cage) and subjected to a 12 hr light/dark cycle with a maintained relative humidity of 60% at 23 °C. The animals were given free access to liquid diet and water. The 48 mice were randomly assigned to 6 groups (*n* = 8) before the commencement of the animal experiment. This animal experiment was reviewed and approved (no.100115) by the Institutional Animal Care and Use Committee (IACUC) of the National Taitung University.

The feed formula of liquid ethanol diet was based on previous studies [36]. The NOR group was fed a Lieber-DeCarli liquid diet (41.4 g casein, 0.5 g L-cystine, 0.3 g DL-methionine, 8.5 g corn oil, 28.4 g olive oil, 2.7 g safflower oil, 115.2 g maltose dextrin, 10 g cellulose, 8.8 g mineral mix, 2.5 g vitamin mix, 0.5 g choline bitartrate, 3 g xanthan gum, and constant volume to 1 L by water; 1000 kcal/L) and given water for 6 weeks. AFLD mice were fed a Lieber-DeCarli liquid ethanol diet (41.4 g casein, 0.5 g L-cystine, 0.3 g DL-methionine, 8.5 g corn oil, 28.4 g olive oil, 2.7 g safflower oil, 25.6 g maltose dextrin, 10 g cellulose, 8.8 g mineral mix, 2.5 g vitamin mix, 0.5 g choline bitartrate, 3 g xanthan gum, 67 mL 95% ethanol, and constant volume to 1 L by water; 1000 kcal/L) and daily given different test substances dissolved in 0.1 mL RO water using oral gavage needle for 6 weeks, respectively [37].

The dosage of red quinoa powder is based on the daily consumption of 25 g of red quinoa for a 60 kg adult. The humans doses were calculated to the dose of mice according to Guidance for Industry Estimating the Maximum Safe Starting Dose in Initial Clinical Trials for Therapeutics in Adult Healthy Volunteers [38]. According to the dose conversion formula (mouse dose (mg/kg bw) = daily adult dose (g)/60 kg*12.3), the RQ-P group was given 5.13 g/kg B.W./day of red quinoa-powder (including rutin 8.46 mg/kg B.W./day). RQB-E and RQB-W are extracted from the bran of red quinoa with different extraction solvents (water or 50% alcohol), therefore, the extracts have different concentrations of rutin. In order to compare the liver protection effect, the RQB-E and RQB-W groups were fed the same dose (1.54 g extract/kg B.W./day). The RQB-E group was given 1.54 g/kg B.W./day of RQB ethanol extract (including rutin 16.4 mg/kg B.W./day. The RQB-W group was given 1.54 g/kg B.W./day of RQB water extract (including rutin 3.92 mg/kg B.W./day). The rutin dose in the rutin group was adjusted according to RQB-E (including 16.4 mg rutin/kg B.W./day). Therefore, the Rutin group was given 16.4 mg/kg B.W./day of rutin. The EtOH group (the negative control) was given water instead of the test sample.

The C57BL/6J mice were euthanized by carbon dioxide. The blood and liver were collected. The liver tissues were rinsed with saline solution and weighed. The second largest lobe of liver tissues were isolated and fixed in 10% of formalin for histopathology. The rest of the liver samples were stored at −80 °C.

### 4.4. Serum Biochemistry Parameters

We determined the activities of alanine transaminase (ALT) (AS101, Randox Laboratories Ltd., Antrim, UK)), aspartate aminotransferase (AST) (AL3801, Randox Laboratories Ltd., Antrim, UK) and alkaline phosphatase (ALP) (AP3802, Randox Laboratories Ltd., Antrim, UK) to indicate liver function and the levels of TC (BXC0261, Fortress Diagnostics Ltd., Antrim, UK) and TG (BXC0271, Fortress Diagnostics Ltd., Antrim, UK) by using the chemistry analyzer (Beckman-700, Fullerton, CA, USA).

### 4.5. Liver Lipids Content

The liver tissue (0.1 g) were ground in 1 mL of ice-cold Folch solution (chloroform/methanol = 2:1; *v/v*) and incubated for 30 min at room temperature. The aqueous layer was aspirated and discarded, and the fixed volume of the organic layer was then evaporated to dryness. The dried lipid layer was dissolved with an equal volume of DMSO and then used to determine the TC (BXC0261, Fortress Diagnostics Ltd., Antrim, UK), TG (BXC0271, Fortress Diagnostics Ltd., Antrim, UK), and glycerol (GY105, Randox Laboratories Ltd., Antrim, UK) levels using commercial assay kit. The cholesterol, triglyceride, and glycerol were used as the standards for the standard curve of TC and TG, and glycerol analysis, respectively.

### 4.6. Lipid Peroxidation and Oxidative Stress in the Liver

The liver tissue was homogenized with phosphate-buffered saline buffer (0.026 M NaCl, 0.0026 M NaH_2_PO_4_, pH 7). The extract was centrifuged at 15,000× *g* for 15 min at 4 °C and then stored at −20 °C before use.

Lipid peroxidation was determined by thiobarbituric acid reactive substances (TBARS) assay. TBARS were quantified on the basis of a standard curve prepared from 1,1,3,3-tetramethoxypropane. The liver lysate (50 µL) was reacted with trichloroacetic acid (300 µL) and 60 mmol/L thiobarbituric acid (100 µL) and then were heated at 95 °C for 30 min. After centrifugation (10,000× *g*, 20 min), and the supernatant of TBARS was measured at 532 nm [39].

The activities of anti-oxidative enzymes were determined by the following commercial kit: SOD assay kit (Ransod, SD125, Randox, Crumlin, Antrim, UK), EnzyChromTM catalase assay kit (ECAT-100, BioAssay Systems, Hayward, CA, USA), GSH-PX assay kit (RANSEL, RS 505, Randox) and EnzyChrom GSH/GSSG Assay Kit (EGTT-100, BioAssay Systems, Hayward, CA, USA).

### 4.7. Hematoxyline and Eosin Stain (H&E Stain)

Fixed-liver tissue was cut into slices of 5 μm thickness. The cell nucleus was stained by hematoxylin and the cytoplasm was stained by eosin in the liver tissue. A section was mounted and sealed with a mounting medium.

### 4.8. Immunoblotting

The liver tissue was homogenized lysis buffer (1% Triton X-100, 20 mM Tris, 40 mM NaF, 0.2% SDS, 0.5% deoxycholate, 1 mM EDTA, 1 mM EGTA, 1 mM Na_3_VO_4_, 100 mM NaCl, pH 7.5). The extract was centrifuged at 15,000× *g* for 15 min at 4 °C and then stored at −20 °C before use. Target protein in liver tissue sample was applied for Western blot according to the previous studies [40,41]. The samples were separated on 10% SDS-PAGE gels and then transferred to polyvinylidene fluoride membranes. PPAR-α polyclonal antibody (sc-398394, Santa Cruz Biotechnology Inc., Dallas, TX, USA), acetyl-CoA carboxylase (ACC) monoclonal antibody (3676, Cell Signaling Technology Inc., Danvers, MA, USA), β-actin monoclonal antibody (MA5-15739, Thermo Fisher Scientific, Rockford, IL, USA) were used to determine the protein expression of PPAR-α, ACC, and β-actin by using immunoblotting.

### 4.9. Statistic Analysis

The data are normally distributed and expressed as the mean ± SD (*n* = 8). Significance between the groups was determined by one-way analysis of variance (ANOVA) followed by Duncan’ s multiple range test by using SPSS 12.0. (SPSS Institute, Inc., Chicago, IL, USA). Differences with *p* < 0.05 were considered statistically significant.

## Figures and Tables

**Table 1:** The extraction yield and rutin content of red quinoa powder and its bran extracts.

Sample	Extract Ratio ^1^ (%)	Rutin (mg/g)
Whole powder (RQ-P)	-	1.65 ± 0.16
Ethanol extract (RQB-E)	14.87	10.65 ± 1.34
Water extract (RQB-W)	19.70	2.45 ± 0.82

^1^ The extract ratio means the ratio of extract weight to the red quinoa weight. RQ-P: red quinoa powder; RQB-E: red quinoa bran ethanol extract; RQB-W: red quinoa bran water extract.

**Table 2:** The body weight, liver weight and liver weight/body weight ratio of the AFLD mice.

Group	Liver Weight (g)	Body Weight (g)	Liver Wight/Body Weight (%)
NOR	0.97 ± 0.09 ^a^	26.63 ± 1.60 ^a^	3.62 ± 0.23 ^b^
EtOH	0.88 ± 0.05 ^b^	23.00 ± 1.31 ^c^	3.83 ± 0.15 ^a^
RQ-P	0.86 ± 0.04 ^b^	22.38 ± 1.19 ^c^	3.85 ± 0.17 ^a^
RQB-E	0.81 ± 0.04 ^b^	23.13 ± 1.25 ^c^	3.52 ± 0.17 ^b^
RQB-W	0.82 ± 0.03 ^b^	23.25 ± 1.39 ^c^	3.53 ± 0.16 ^b^
Rutin	0.85 ± 0.08 ^b^	24.75 ± 1.49 ^b^	3.44 ± 0.17 ^b^

The data are presented as the means ± SD (*n* = 8). The means followed by the same letter within each column did not differ significantly from each other (*p* > 0.05). However, the means followed by different letter expressed significant difference from each other (*p* < 0.05). Two groups of mice were fed with normal liquid diet (NOR group) or ethanol liquid diet (EtOH group) without the administration of test materials, respectively. The other mice fed ethanol liquid diet were administrated with red quinoa powder (5.13 g/kg B.W./day, RQ-P group), red quinoa bran ethanol extracts (1.54 g/kg B.W./day, RQB-E group), red quinoa bran water extracts (1.54 g/kg B.W./day, RQB-W group), and rutin (16.4 mg/kg B.W./day, Rutin group).

**Table 3:** Serum AST, ALT, and ALP activities of the AFLD mice.

Group	AST Activity (U/L)	ALT Activity (U/L)	ALP Activity (IU/L)
NOR	40.5 ± 3.8 ^b^	14.5 ± 0.5 ^d^	65.0 ± 2.0 ^c^
EtOH	64.5 ± 18.7 ^a^	23.8 ± 5.3 ^a^	93.9 ± 11.7 ^a^
RQ-P	46.4 ± 4.9 ^b^	18.0 ± 2.1 ^bc^	100.4 ± 7.0 ^a^
RQB-E	40.0 ± 2.0 ^b^	15.9 ± 1.4 ^cd^	73.3 ± 4.9 ^b^
RQB-W	44.1 ± 2.9 ^b^	18.9 ± 1.6 ^bc^	79.3 ± 6.4 ^b^
Rutin	47.6 ± 2.4 ^b^	19.6 ± 4.2 ^b^	79.4 ± 5.0 ^b^

The data are presented as the means ± SD (*n* = 8). The means followed by the same letter within each column did not differ significantly from each other (*p* > 0.05). However, the means followed by different letter expressed significant difference in each other (*p* < 0.05). Two groups of mice were fed with normal liquid diet (NOR group) or ethanol liquid diet (EtOH group) without the administration of test materials, respectively. The other mice fed ethanol liquid diet were administrated with red quinoa powder (5.13 g/kg B.W./day, RQ-P group), red quinoa bran ethanol extracts (1.54 g/kg B.W./day, RQB-E group), red quinoa bran water extracts (1.54 g/kg B.W./day, RQB-W group), and rutin (16.4 mg/kg B.W./day, Rutin group). AST: aspartate aminotransferase; ALT: alanine aminotransferase; ALP: alkaline phosphatase.

**Table 4:** Serum and liver lipid contents of the AFLD mice.

Group	Serum TC (mg/dL)	Serum TG (mg/dL)	Liver TC (mg/g)	Liver TG (mg/g)	Liver Glycerol (mg/g)
NOR	107.1 ± 8.4 ^cd^	84.3 ± 13.9 ^b^	1.51 ± 0.14 ^ab^	0.95 ± 0.24 ^ab^	0.70 ± 0.15 ^b^
EtOH	128.8 ± 8.6 ^a^	168.8 ± 21.2 ^a^	1.42 ± 0.08 ^b^	1.13 ± 0.42 ^a^	1.08 ± 0.18 ^a^
RQ-P	83.1 ± 3.1 ^e^	54.5 ± 6.3 ^d^	1.50 ± 0.04 ^ab^	0.60 ± 0.14 ^c^	0.40 ± 0.10 ^c^
RQB-E	100.9 ± 8.1 ^d^	68.3 ± 3.5 ^c^	1.60 ± 0.13 ^a^	0.80 ± 0.12 ^bc^	0.44 ± 0.08 ^c^
RQB-W	110.1 ± 7.0 ^bc^	75.4 ± 8.7 ^bc^	1.50 ± 0.07 ^ab^	0.94 ± 0.18 ^ab^	0.74 ± 0.15 ^b^
Rutin	116.3 ± 10.9 ^b^	89.0 ± 14.9 ^b^	1.44 ± 0.06 ^b^	0.93 ± 0.23 ^ab^	0.70 ± 0.16 ^b^

The data are presented as the means ± SD (*n* = 8). The means followed by the same letter within each column did not differ significantly from each other (*p* > 0.05). However, the means followed by different letter expressed significant difference from each other (*p* < 0.05). Two groups of mice were fed with normal liquid diet (NOR group) or ethanol liquid diet (EtOH group) without the administration of test materials, respectively. The other mice fed ethanol liquid diet were administrated with red quinoa powder (5.13 g/kg B.W./day, RQ-P group), red quinoa bran ethanol extracts (1.54 g/kg B.W./day, RQB-E group), red quinoa bran water extracts (1.54 g/kg B.W./day, RQB-W group), and rutin (16.4 mg/kg B.W./day, Rutin group). TG: triglyceride; TC: total cholesterol.

## Data Availability

The data presented in this study are available on request from the corresponding author. The data are not publicly available due to ethical restriction.

## Sample Availability

Samples of the red quinoa are available from the authors.
